# Development of Live Vaccine Candidates for Canine Influenza H3N2 Using Naturally Truncated NS1 Gene

**DOI:** 10.1155/2024/4335836

**Published:** 2024-03-22

**Authors:** Jaehyun Hwang, Sun-Woo Yoon, Eulhae Ga, Suyun Moon, Jaeseok Choi, Eunseo Bae, Jung-Ah Kang, Hye Kwon Kim, Dae Gwin Jeong, Daesub Song, Woonsung Na

**Affiliations:** ^1^College of Veterinary Medicine, Chonnam National University, Gwangju 61186, Republic of Korea; ^2^Department of Biological Sciences and Biotechnology, Andong National University, Andong 36729, Republic of Korea; ^3^Bionanotechnology Research Center, Korea Research Institute of Bioscience and Biotechnology, Daejeon 34141, Republic of Korea; ^4^Department of Biological Sciences and Biotechnology, College of National Sciences, Chungbuk National University, Cheongju 28644, Republic of Korea; ^5^College of Veterinary Medicine and Research Institute for Veterinary Science, Seoul National University, Seoul 08826, Republic of Korea; ^6^Department of Oral Microbiology and Immunology and Dental Research Institute, School of Dentistry, Seoul National University, Seoul 03080, Republic of Korea

## Abstract

The NS1 influenza protein of influenza A virus is a viral nonstructural protein encoded by the NS gene segment that has multiple accessory functions during viral infection. In recent years, the major role ascribed to NS1 has been its inhibition of host immune responses, especially the limitation of both interferon (IFN) production and the antiviral effects of IFN-induced protein. We isolated an equine influenza virus with a naturally truncated NS1 gene in our previous study. In this current research, we inserted this partially truncated NS gene into the H_3_N_2_ canine influenza virus using reverse genetics to develop a live attenuated vaccine strain. To evaluate whether the developed strain is suitable as a live vaccine candidate, we compared its replication kinetics with wild-type virus in MDCK cells and specific pathogen-free eggs. Additionally, we investigated host antiviral gene expression, viral replication in the respiratory system, and associated lung tissue damage in mice experiments. To confirm the efficacy of the vaccine candidate, we evaluated the immunogenicity and protectivity of the developed vaccine strain against canine influenza H_3_N_2_, compared with a commercial inactivated vaccine. Through these experiments, it was confirmed that the naturally truncated NS1 inserted virus has sufficient potential as a live vaccine candidate, and we hopefully expect that this study would make a great contribution to the development of a live vaccine for canine influenza H_3_N_2_.

## 1. Introduction

Influenza virus is a member of the family Orthomyxoviridae [1] and a causative agent of respiratory disease in various species. Influenza viruses are divided into four genera: A, B, C, and D. Influenza A virus infects a wide range of avian and mammalian hosts, including human, while influenza B and C infects human hosts, and influenza D infects cattle and pigs [2], respectively. The natural host of the influenza virus is wild birds, and mutated viruses are transmitted to other species through the adaptation of a new host.

In 2007, in Korea, it was reported that the avian influenza virus transmitted from poultry to dogs, and since then, the canine influenza virus (CIV) spread worldwide as well as continually circulated in the dog population. CIV is divided into two subtypes: H_3_N_2_ and H_3_N_8_ [1, 3–5], each originated from avian H_3_N_2_ and equine H_3_N_8_, respectively. The H_3_N_8_ subtype was first isolated in January 2004 from racing greyhounds affected with respiratory disease in Florida [4]. This virus continuously circulated among the dog population in the United States for more than a decade, with most occurring in Colorado and the Northeast states after about 2005. However, after 2012, the outbreak was restricted to a few animal shelters only. By 2016, it was eventually reduced to very low levels [6]. Avian-originated H_3_N_2_ CIV, which was first reported in 2007 from dogs exhibiting severe respiratory disease in Korea [3]. According to the subsequent study of serological analysis, this H_3_N_2_ CIV has been already existed in Korea from 2005 and has existed in China from 2006 [7]. This type of virus has been continuously reported from dogs exhibiting respiratory clinical signs in both China and Korea. It has been circulated stably in the dog population in Asia [8–12]. In February–March 2015, a dog infected with the prevalent CIV in South Korea was adopted and brought to Chicago, leading to the spread of the virus around the state. Despite local quarantine policy, it has spread to other several states of the United States, particularly Georgia and North Carolina [13].

Influenza viruses have a negative sense eight-segment single-stranded RNA genome, which encodes 11 proteins exhibiting different important roles about the viral life cycle. Among 11 proteins, nonstructural protein1 (NS1 protein) plays a key role in infection through a multitude of molecular interaction partners, host cell activities, and structural plasticity or polymorphism [14]. According to previous studies that analyzed roles of NS gene using recombinant or truncated NS genes [15–17], partially truncated NS1 protein has reduced activation of Akt kinase, whereas it increased phosphorylation of interferon regulatory factor 3 (IRF3) in cells and it showed dramatically attenuation of virulence in mice. The partially truncated NS1 protein expresses an impaired function of inhibiting host antiviral responses, resulting in the attenuation of viral replication.

Currently, the vaccines that protect against CIV are in the inactivated form and are thought to have some limitations in protecting against the influenza virus that circulates and mutates in the dog population. We conducted this experiment to solve these limitations and to develop a live vaccine with higher efficacy and excellent cross-protection effect.

In this study, we inserted the naturally truncated NS1 gene into CIV H_3_N_2_ using reverse genetics technology and verified the successful replacement of the gene. This live vaccine candidate strain was confirmed to be suitable for vaccine production in mammalian cells and specific pathogen-free (SPF) eggs and induced a higher immunogenicity and attenuation than wild-type virus in mice experiments. In addition, we also have found that the vaccine candidate exhibits superior immune response and better protection against wild-type viruses compared to commercial inactivated vaccines in mice experiments. Put all of these results together, this developed strain was confirmed as a possible live vaccine candidate for canine influenza H_3_N_2_ and expected to be helpful to form a live vaccine development platform using naturally truncated NS1 gene and also develop a successful canine influenza live vaccine.

## 2. Materials and Methods

### 2.1. Experimental Animals

In total, 207 SPF female BALB/c mice were purchased from Dae Han Bio Link (Eumseong, South Korea). All of the experiments with animals and viruses were conducted in biosafety level 2 facilities at Chonnam National University.

### 2.2. Cells and Viruses

Madin–Darby canine kidney cells and human embryonic kidney 293T cells were purchased from the American Type Culture Collection (ATCC). MDCK cells and human 293T cells were grown in DMEM and Opti-MEM containing 5% fetal bovine serum, and 1% antibiotics, respectively, and the incubator condition was maintained at 37°C and 5% CO_2_. The A/equine/Kyonggi/SA1/2011 (SA1) and A/canine/Korea/01/2007 (CIV) viruses were cultured in the allantoic cavity of 10-day-old embryonated chicken eggs, and CIV with naturally truncated NS gene of SA1 virus (CIV^SA1^) produced through the reverse genetics system was also cultured under the same conditions. These viruses were stored in an 80°C deep freezer until used for the experiment.

### 2.3. Generation of Recombinant Virus

Viral RNA was extracted from the allantoic fluid using a QIAamp Viral RNA Mini Kit (Qiagen Inc., CA, USA). The RNA of the SA1 virus was reverse transcribed, and the NS gene was amplified by polymerase chain reaction (PCR) using a OneStep reverse transcription PCR kit (Qiagen Inc.) with universal primers and then cloned into a pHW2000 plasmid, as described previously [18]. We used the plasmid-based reverse genetics system to design recombinant CIV with the truncated NS gene.

To sum up briefly, eight pHW2000 plasmids containing each of eight segments of influenza A virus genes were transfected into cocultured cells of MDCK and 293T cells. The recombinant CIV with the naturally truncated NS gene (CIV^SA1^) was rescued from the supernatant of the cocultured cells. The rescued virus was inoculated in 10-day-old embryonated chicken eggs for propagation. After the generation of the recombinant virus, we verified the full genome sequence of this recombinant virus using RT-PCR as previously described [19]. The virus was stored at −80°C before using for the experiment.

### 2.4. Viral Replication Kinetics

MDCK cells were cultured in six well-tissue culture plates the day before the virus inoculation. The 0.001 multiplicity of infection (MOI) of CIV and CIV^SA1^ viruses were adsorbed to the cells for 1 hr in an incubator at 37°C and 5% CO_2_. After removing the inoculum, the cells were washed with PBS and then maintained with influenza media (MEM containing 1 *μ*g/ml TPCK-treated Trypsin). The 10-day-old embryonated chicken eggs were incubated at 37°C and 5% CO_2_. The CIV and CIV^SA1^ viruses were infected to the allantoic cavity of the SPF eggs. The viral titer of those viruses was 10^4^ EID_50_ (50% of tissue culture infectious dose). The supernatants from three infected wells and three eggs were collected at indicated time points, and stored at −80°C until used for the experiment. The viral replication kinetics were analyzed using TCID_50_ assay. The TCID_50_ assay was conducted as previously described [20]. Briefly, 1 day before the experiment, MDCK cells were seeded at a concentration of 2 × 10^5^ cells/ml in a 96-well plate. The desired virus was diluted tenfold in an influenza media, and 100 *μ*l of each dilution was added to five wells. After maintaining it for 1 hr in the incubator, the inoculum was removed, and the cells were washed with PBS. Then, 200 *μ*l of influenza media was inoculated to cells, and the plates were maintained in the incubator for 3 days. Afterward, wells showing cytopathic effects were considered positive. The TCID_50_ was calculated by the method of Reed and Muench as described elsewhere [21].

### 2.5. Analysis of Host Antiviral Signals and T-Cell Immunity through Mice Experiments

To compare the host antiviral gene expression related to viral NS1 protein (RIG-I and its downstream genes; IFN-*β*, ISG15, IRF7, RANTES, IL-6, and IP-10) and T-cell immune induction of CIV and CIV^SA1^ viruses, 7 weeks-old female BALB/c 36 mice were used [16, 22]. Twelve mice were intranasally infected with PBS, CIV, and CIV^SA1^ viruses, respectively. The viral infectious dose was 10^5^ EID_50_/mouse. Euthanasia was performed on each three mice on the 3rd, 5th, 7th, and 9th day postinfection (dpi) to obtain lungs and spleen. The viral infectious dose was 10^5^EID_50_/mouse. Among the obtained samples, the right lobes of the lung were used for measuring the mRNA expression level of genes related to RIG-I and downstream of RIG-I (IFN-*β*, IL-6, IP-10, ISG15, IRF-7, and RANTES), and the left lobes of the lung and spleen were used for the evaluation of T-cell immune induction capability. RNA was extracted from mice right lobes using RNeasy Mini Kit (Qiagen) according to the manufacturer's protocol. Reverse transcription was performed using 1 *μ*g of total RNA with TOPscript^TM^ RT DryMIX (Enzynomics). Realtime PCR was performed with TOPreal™ SYBR Green qPCR High-ROX PreMIX (Enzynomics), and the relative expression of the target gene was analyzed using the Pfaffl method [23]. The GAPDH housekeeping gene was used as an endogenous standard gene. The primers used in this study are documented in Table 1. T-cell immune induction capability was analyzed using ELISPOT and FACS assay as previously described [24].

### 2.6. Comparison of Viral Replication in Mice

To compare in vivo viral replication rate, 7-week-old female BALB/c 23 mice were used. Nine mice were intranasally infected with CIV and CIV^SA1^ viruses, respectively, and five mice were inoculated with PBS. The viral infectious dose was 10^6.5^ EID_50_/mouse. At dpi 4, euthanasia was performed on nine mice of the virus-infected groups and five mice of the PBS-inoculated group, respectively, and lung and nasal wash samples were collected. The left lobes of the lung were homogenized to analyze the residual viral titer in the lower respiratory tract, and the nasal wash samples were used to analyze the residual viral titer in the upper respiratory tract. As an analysis method, TCID_50_ assay was used and calculated using the Reed–Muench method.

### 2.7. Histopathology and Immunohistochemistry (IHC) Examination

The remaining right lobes of the lung from evaluation of viral replication in mice experiment were fixed with 10% neutral buffered formalin at room temperature for 48 hr. Four micrometer-thick sections were prepared from the paraffin-embedded tissues. The sections were stained as previously described for histopathology and IHC examination using hematoxylin and eosin (H&E) and influenza A NP-specific polyclonal rabbit anti-sera (Invitrogen, cat #PA5-32242), respectively [8, 25]. Histopathological lesions of lung samples were evaluated in five categories: necrosis of bronchial epithelial cells, peribronchial and perivascular cuffing, inflammatory cell infiltration in the alveoli, alveolar hemorrhage, and atelectasis. Histopathology and IHC examination were graded on a scale of 0–4, according to the severity of lung lesions (Table S1).

### 2.8. Evaluation of Humoral and Cellular Immunity and Protection against Wild-Type Virus

**Figure 1:**
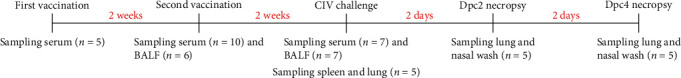
Diagram of experiment schedule for vaccine efficacy evaluation.

To compare the efficacy of commercial inactivated vaccines and the live vaccine candidate, 6-week-old female BALB/c mice were divided into six groups, and experiments were performed as described in Figure 1 and Table 2. Vaccines were standardized by hemagglutinin units (HAU) and inoculated twice at 2-week intervals. Serum was obtained through orbital blood sampling at three time points: before 1st vaccination, 2 weeks after the 1st vaccination, 2 weeks after the 2nd vaccination, and Broncho–Alveolar Lavage Fluid (BALF) was collected at two time points: 2 weeks after the 1st and 2nd vaccination. Spleens and lungs were obtained after euthanasia on 2 weeks after the 2nd vaccination. At that time, a challenge was performed with wild-type virus, CIV of 10^6.5^EID_50_/mouse, and lungs and nasal washes were obtained after euthanasia on day postchallenge (dpc) 2 and 4. From the obtained serum, humoral immunity was analyzed through IgG enzyme-linked immunosorbent assay (ELISA), serum neutralization (SN), and hemagglutination inhibition (HI) assay. From the collected BALF, mucosal immunity was analyzed through IgA ELISA. The IgG and IgA ELISA were performed using CIV whole virus particles coated as the antigen. The procedure for ELISA, SN, and HI assay were carried out as previously described [26, 27]. From lungs and spleen of euthanasia mice on 2 weeks after the 2nd vaccination, cellular immunity was analyzed through mouse IFN-*γ* enzyme-linked immunosorbent spot (ELISpot) assay using BD™ ELISPOT Mouse IFN-*γ* kit. The collected lungs and spleens were isolated into single cells. The splenocytes and pneumocytes were seeded at a concentration of 10^6^ cells per well and treated with 10^5.5^ EID/100 *μ*l of CIV. The cells were incubated overnight at 37°C and 5% CO_2_. Subsequently, the experiment was conducted following the manufacturer's manual. From the lung and nasal wash samples of euthanasia mice on dpc 2 and 4, the protection against wild-type virus was analyzed through TCID_50_ assay.

### 2.9. Statistical Analysis

The data from each experiment were plotted as mean values ± standard deviation (SD). Differences between mean values of indicated groups were determined by a two-tailed Student's *t* test or one-way analysis of variance (one-way ANOVA) with Dunnett's multiple comparisons test. If the *P* value for that difference is less than 0.05, the result was considered statistically significant ( ^*∗*^*P* ≤ 0.05,  ^*∗∗*^*P* ≤ 0.01,  ^*∗∗∗*^*P* ≤ 0.001).

## 3. Results

### 3.1. Generation and Characterization of CIV^SA1^ Live Attenuated Influenza Vaccine (LAIV)

**Figure 2:**
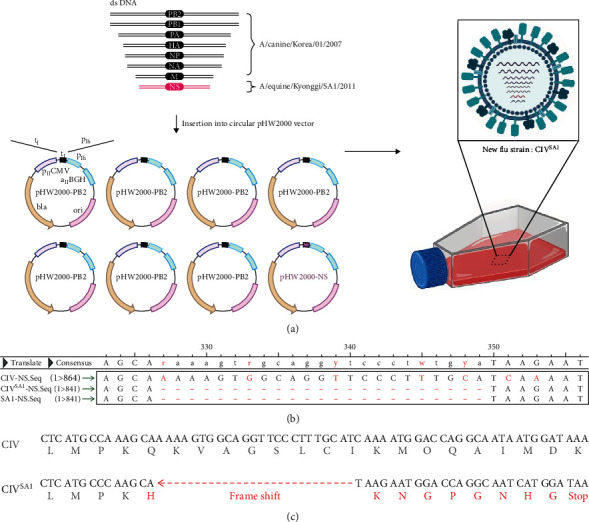
Generation of CIV^SA1^ LAIV by reverse genetics: NS gene of A/equine/Kyonggi/SA1/2011 (SA1) was introduced into A/canine/Korea/01/2007 to generate CIV^SA1^ LAIV by using plasmid-based reverse genetic technology (a). SANGER sequence analysis confirmed that the NS gene of CIV^SA1^ was identical with that of SA1 (b). The 23-nt deletion results in a frame shift, thereby, generating in-frame early stop codon (c).

In a previous study, we isolated A/canine/Korea/01/2007 (CIV) and A/equine/Kyonggi/SA1/2011 (SA1) and also found that when the NS gene of SA1 is inserted into human H1N1 influenza A virus (A/Puerto Rico/8/1934), viral virulence was attenuated in mice[3, 15]. To develop CIV LAIV, reverse genetics technology was used to generate CIV^SA1^ strain. The NS gene of CIV was replaced with that of SA1, and all other seven genes were composed of genes from CIV (Figure 2(a)). All eight gene sequences of CIV^SA1^ strain were verified by Sanger DNA sequencing. we confirmed that the NS gene sequence of CIV^SA1^ and SA1 were identical and the NS1 sequence of LAIV has a 327–349 deletion (Figure 2(b)). Thereby, the frameshift generates a premature stop codon and truncated NS1 protein (Figure 2(c)). Through the results above, it was confirmed that CIV^SA1^ strain, cH_3_N_2_ with naturally truncated NS gene, was successfully generated.

### 3.2. Viral Replication Kinetics

**Figure 3:**
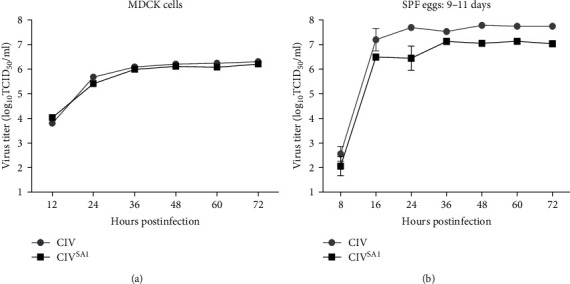
Growth kinetics of CIV and CIV^SA1^ in MDCK cells and SPF eggs. MDCK cells were infected with the CIV or CIV^SA1^ virus at an MOI of 0.001 (a) and SPF eggs of 9–11 days were infected with the CIV or CIV^SA1^ virus at 10^4^ EID_50_/egg (b). The viral supernatants were collected at the indicated time points. The viral titers of supernatant samples were titrated with TCID_50_ method. Data are shown as the mean ± standard deviation from three independent experiments. Statistical significance was calculated using two-tailed Student's t test.

To confirm whether the replication of CIV^SA1^ strain was inhibited in MDCK cells and SPF eggs, which were used for influenza vaccine production, the viral replication kinetics of CIV and CIV^SA1^ were compared in MDCK cells and SPF eggs. The viral titers of CIV^SA1^ in MDCK cells and SPF eggs were tended to be slightly lower than that of CIV after 24 hr, but there was no statistical difference between the titer of two viruses (Figure 3). According to this result, CIV^SA1^, LAIV candidate, is considered suitable for vaccine production in MDCK cell line and SPF eggs.

### 3.3. Antiviral Responses of Mice Are Upregulated under CIV^SA1^ Infection

**Figure 4:**
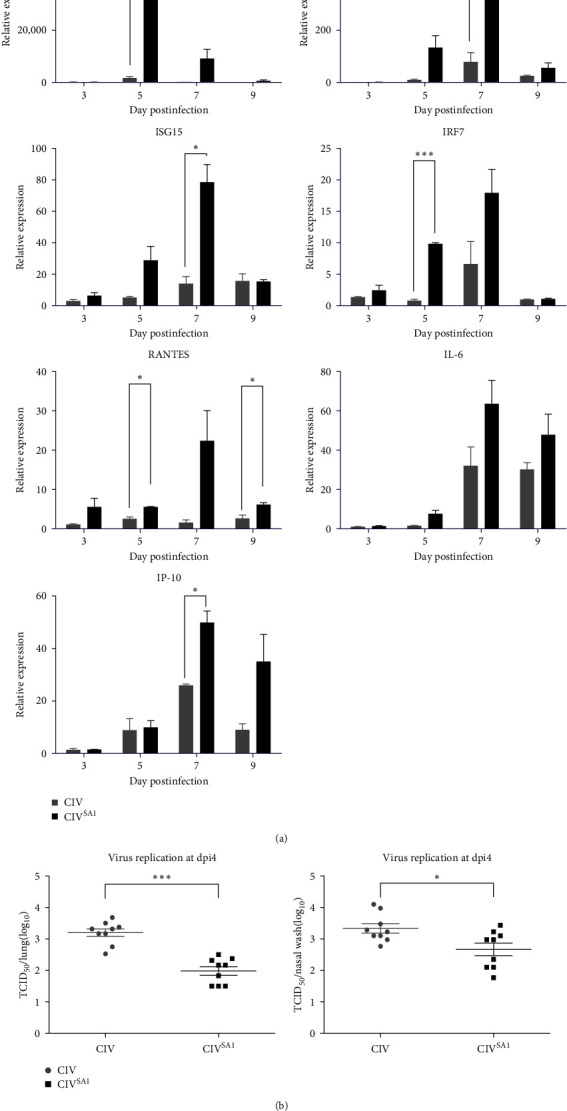
Antiviral gene mRNA levels and viral replication in lung from mice infected with CIV or CIV^SA1^. Mice were intranasally inoculated with CIV (*n* = 12), CIV^SA1^ (*n* = 12), and PBS (*n* = 12), respectively. The viral infectious dose was 10^5^ EID_50_/mouse. Euthanization was performed on each three mice to collect lungs on dpi 3, 5, 7, and 9. The mRNA expression levels in those lungs were measured by real-time PCR and analyzed using the Pfaffl method. The GAPDH housekeeping gene was used as an endogenous standard gene (a). Mice were intranasally infected with CIV (*n* = 9), CIV^SA1^ (*n* = 9), and PBS (*n* = 5), respectively. The viral infectious dose was 10^6.5^ EID_50_/mouse. The viral titer of lung and nasal wash samples from dpi 4 euthanized mice was measured by TCID_50_ method (b). The results were expressed as mean ± SD and Statistical significance was calculated using two-tailed Student's t test ( ^*∗*^*P* ≤ 0.05,  ^*∗∗*^*P* ≤ 0.01,  ^*∗∗∗*^*P* ≤ 0.001).

Next, we investigated the downstream genes following RIG-I activation and innate immune response in mice infected with CIV^SA1^ virus. Mice were infected with CIV and CIV^SA1^ at 10^5^ EID_50_ per mouse. The mRNA levels of the downstream genes were determined using realtime PCR at dpi 3, 5, 7, and 9. As shown in Figure 4(a), the expression levels of RIG-I mRNA in CIV^SA1^-infected mice were 27 times higher than those in CIV-infected mice. Accordingly, the RIG-I downstream genes, IFN-*β*, ISG15, IRF7, RANTES, IL-6, and IP-10, were all increased. These data show that the deletion of the NS1 effector domain increases the expression level of viral-induced RIG-I mRNA in mice and, accordingly, the amount of host antiviral gene expressions.

According to the above results, we compared residual viral titers in the upper and lower respiratory tracts of mice, infected with CIV or CIV^SA1^ viruses. In both lung and nasal wash samples from CIV^SA1^-infected mice on dpi 4, it was confirmed that viral titers were significantly lowered compared with those from CIV-infected mice (Figure 4(b)). Summarizing all of the results above, the virus with naturally truncated NS gene is unable to evade the host antiviral responses, resulting in reduced viral replication in the mice lungs.

### 3.4. Pulmonary Histopathology and Immunohistopathology in Mice Inoculated with CIV^SA1^

**Figure 5:**
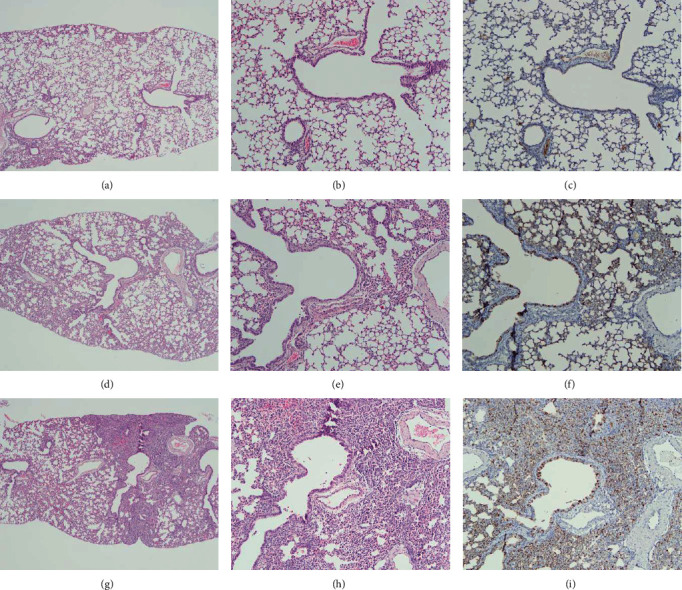
Lung histopathology of mice infected with CIV and CIV^SA1^. Mice were inoculated with PBS (a–c), CIV^SA1^ (d–f), and CIV (g–i) at a dose of 10^6.5^ EID_50_. The H&E (a, b, d, e, g, and h) and IHC (c, f, and i) staining were performed on mice lung tissues collected on dpi 4. Images are representative of three mice per group and obtained at 40x magnification (a, d, and g) and 100x (b, c, e, f, h, and i).

To examine viral pathology, we performed H&E and IHC staining on all of lung tissues collected from three and seven mice on dpi 2 and 4, respectively. As shown in Table S2, “Peribronchial and perivascular cuffing” and “Alveolar hemorrhage” in lungs of CIV^SA1^-infected mice showed similar scores with that of CIV-infected mice in H&E staining samples on dpi 4. However, “Inflammatory cell infiltration in the alveoli” and “Atelectasis” in lungs of CIV^SA1^-infected mice showed lower scores than that of CIV-infected mice. The IHC scores in lungs of CIV^SA1^-infected mice were one core lower than that of CIV-infected mice. The total score of H&E and IHC in lungs of CIV^SA1^-infected mice was lower than that of CIV-infected mice (Figure S1). As shown in Figure 5, the lungs of CIV^SA1^-infected mice exhibit histopathological features that are closer to normal compared to that of CIV-infected mice. Mice infected with CIV exhibited mild atelectasis characterized with alveolar hemorrhage and inflammatory cell infiltration in the alveoli. The number of NP antibody-positive pulmonary cells from CIV-infected mice was more than that from CIV^SA1^-infected mice. In summary, combining the histopathological and IHC results, it can be concluded that infection of mice with CIV^SA1^ not only leads to a decrease in viral replication compared to CIV infection but also reduces the actual lung damages.

### 3.5. Cellular Immunity in Mice Inoculated with CIV^SA1^

**Figure 6:**
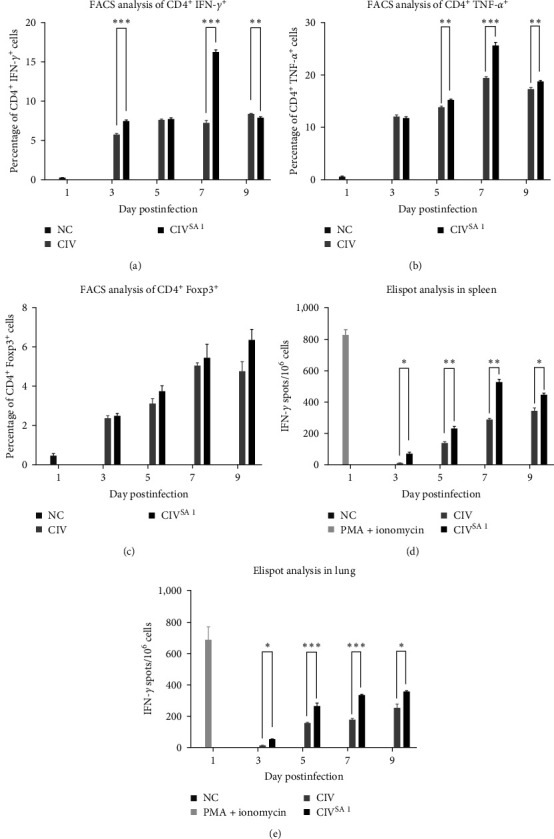
Analysis of cellular immunity in both spleen and lung of mice inoculated with CIV or CIV^SA1^. Mice were inoculated with CIV (*n* = 12), CIV^SA1^ (*n* = 12), and PBS (*n* = 12), respectively. The viral infectious dose was 10^5^ EID_50_/mouse. Euthanize was performed on each three mice to collect lungs on dpi 3, 5, 7, and 9. FACS was performed in spleen to determine the percentage of CD4^+^IFN-*γ*^+^ cells (a), CD4^+^TNF-*α*^+^ cells (b), and CD4^+^Foxp3^+^ cells (c). ELISPOT assay for IFN-*γ* was performed on collected spleens (d) and lungs (e). The results were expressed as the mean ± SD. Statistical significance was calculated using two-tailed Student's t test ( ^*∗*^*P* ≤ 0.05,  ^*∗∗*^*P* ≤ 0.01,  ^*∗∗∗*^*P* ≤ 0.001).

To examine cellular immunity in mice inoculated with CIV^SA1^, mice were inoculated with CIV and CIV^SA1^ at a dose of 10^5^ EID_50_. The lungs and spleens of the mice euthanized at dpi 3, 5, 7, and 9 were collected and were used to investigate cellular immunity by ELISPOT and FACS. Based on the FACS results, we found that the levels of CD4^+^ IFN-*γ*^+^ cells in CIV^SA1^-infected mice were significantly higher on dpi 3 and 7 (Figure 6(a))). Additionally, the levels of CD4^+^TNF-*α*^+^ cells in CIV^SA1^-infected mice were significantly higher on dpi 3, 5, and 7 (Figure 6(b)). Although no significant difference was detected in CD4^+^Foxp3^+^ cells, there was a trend of higher distribution in CIV^SA1^-infected mice (Figure 6(c)). These FACS data showed that, in mice inoculated with naturally NS truncated virus, type 1 helper T cells and regulatory T cells showed a tendency to be more activated than in mice inoculated with wild-type virus. Based on the ELISPOT results, the secreted IFN-*γ* proteins were significantly upregulated in both spleen and lung cells collected from the CIV^SA1^-inoculated mice on dpi 3, 5, 7, and 9 (Figures 6(d) and 6(e)). These ELISPOT data showed that the total magnitude of the antigen-specific Th1 cells and CD8^+^ cells in both spleen and lung of CIV^SA1^-inoculated mice were significantly upregulated than that of CIV-inoculated mice. Moreover, the level of IFN-*γ* spots from spleen and lung samples tends to steadily increase until dpi 7.

### 3.6. Humoral, Mucosal, and Cellular Immunity and Protective Efficacy Induced by CIV^SA1^, Live Attenuated Vaccine Candidate

**Figure 7:**
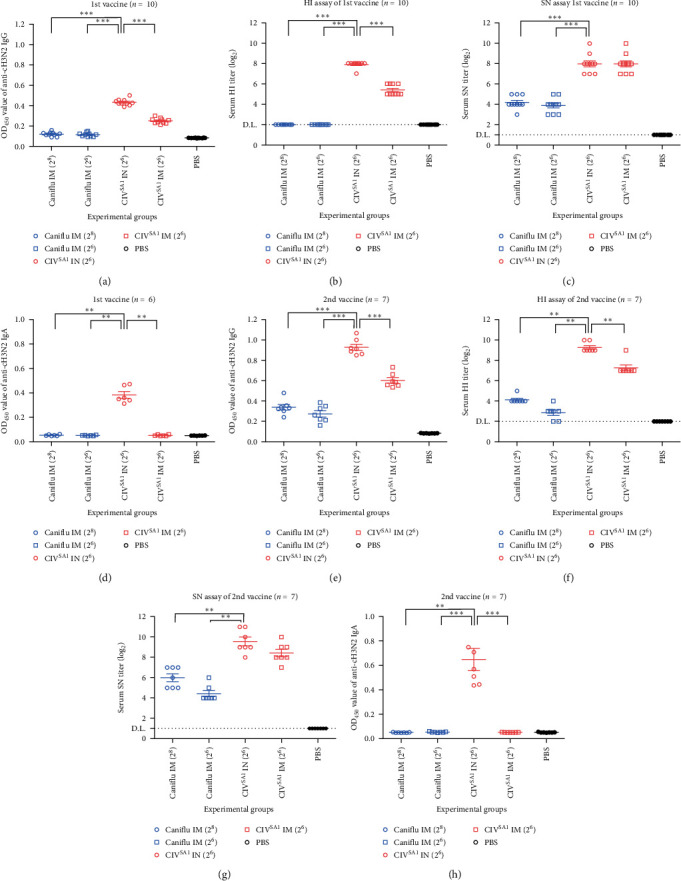
Determining both humoral and mucosal antibody titers against CIV. ELISA assay was performed to measure the serum IgG (a and e) and BALF IgA (d and h) antibody titers. The O.D. values were measured at 450 nm wavelength. The serum HI (b and f) and SN (c and g) antibody titer were analyzed using CIV whole virus. Serum and BALF samples were collected from mice at 2 weeks after the 1st (a–d) and 2nd (e–h) vaccine. The OD450 values of 1 : 2^13^ dilution of serum IgG antibody samples (a and e) and 1 : 2^3^ dilution of BALF IgA antibody samples (d and h) were compared among the different groups. The results were expressed as the mean ± SD, and the number of samples for each experiment was indicated in the respective figures. Statistical significance was calculated using a one-way analysis of variance (one-way ANOVA). ( ^*∗∗*^*P* ≤ 0.01,  ^*∗∗∗*^*P* ≤ 0.001).

**Figure 8:**
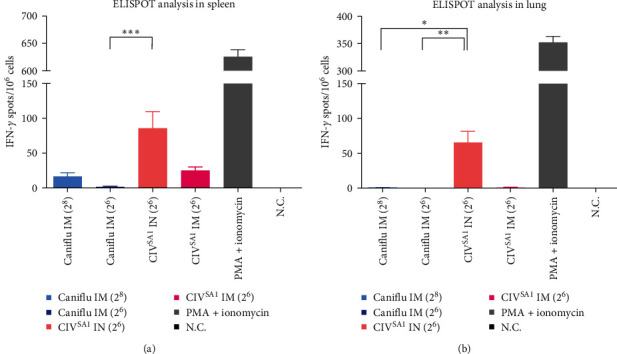
Determining cellular immunity against CIV. In the 2nd week after the 2nd vaccination, mice were euthanized to obtain the spleen (*n* = 5) and lungs (*n* = 5). The acquired spleen and lung tissues were subsequently separated into single cells. Following treatment with CIV at a dose of 10^5.5^ EID/100 *μ*l for splenocytes (a) and pneumocytes (b), mouse IFN-*γ* ELISPOT analyses were conducted. The results were expressed as the mean ± SD. Statistical significance was calculated using a one-way analysis of variance (one-way ANOVA) ( ^*∗*^*P* ≤ 0.05,  ^*∗∗*^*P* ≤ 0.01,  ^*∗∗∗*^*P* ≤ 0.001).

To determine the possibility of the CIV^SA1^ virus as a live attenuated vaccine, a mice experiment was conducted as described in Figure 1 and Table 2. Mice were vaccinated twice with each vaccine at 2 weeks intervals and challenged with wild-type virus, CIV after 2 weeks from 2nd vaccination. The dose of inoculated vaccines was standardized as HAU, and the vaccine inoculation and viral challenging were performed as described (Figure 1 and Table 2). To examine humoral immunity, the serums of mice were collected through retro-orbital at 1 day before 1st vaccination, 2nd vaccination, and challenge. The serum IgG antibody titers of 1st and 2nd vaccine after 2 weeks, specifically targeting the CIV virus, were significantly higher in the CIV^SA1^ IN group, compared to that of other groups (Figures 7(a) and 7(e) and Figure S2). Moreover, the CIV^SA1^ IN group showed significantly higher levels of HI titer, representing antibody titers specific to HA protein, and SN titers, representing antibody titers that inhibit virus replication in cells, compared to the Caniflu-max vaccination group. The HI titers of the CIV^SA1^ IN group were significantly higher than those of the CIV^SA1^ IM group (Figures 7(b) and 7(f)). Although no significant difference was observed in SN titers, there was a tendency for SN titer of 2nd vaccine serum in CIV^SA1^ IN group to be higher than that in the CIV^SA1^ IM group (Figures 7(c) and 7(g)). The comparative results of mucosal IgA antibody production capacity revealed that only the CIV^SA1^ IN group exhibited the generation of mucosal IgA antibodies (Figures 7(d) and 7(h) and Figure S2). To compare the differences in cellular immune induction among vaccinated groups, five mice per group were euthanized in the 2 weeks after the 2nd vaccination. Spleens and lungs were collected, and mouse IFN-*γ* ELISPOT assay were conducted on the splenocytes and pneumocytes (Figure 8). The results showed that the CIV^SA1^ IN and CIV^SA1^ IM groups exhibited a higher number of IFN-*γ* spots in both the spleens and lungs, compared to the Caniflu IM groups. Among the live vaccine groups, the intranasal route induced a higher number of IFN-*γ* spots. In the spleen, the CIV^SA1^ IN group showed a significantly higher number of IFN-*γ* spots than the Caniflu IM (2^6^) group, while in the lungs, the CIV^SA1^ IN group exhibited significantly more IFN-*γ* spots than the Caniflu IM (2^8^) and Canilflu IM (2^6^) groups. Summarizing the above results, CIV^SA1^, LAIV vaccine candidate, induced significantly higher humoral, mucosal, and cellular immunity compared with the commercial inactivated vaccine. Additionally, the intranasal vaccine route induces higher humoral, mucosal, and cellular immunity compared to the intramuscular vaccine route.

**Figure 9:**
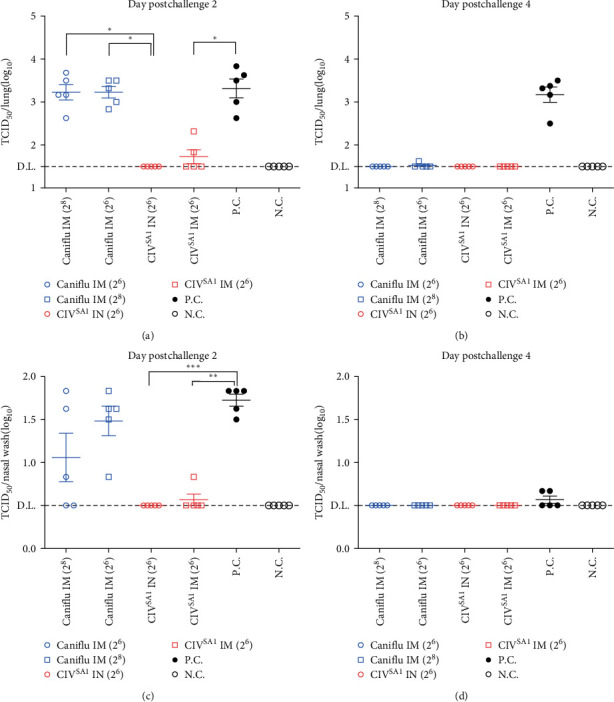
Protection efficacy of mice vaccinated with commercial vaccine or CIV^SA1^. The vaccinated or PBS-inoculated mice were challenged with CIV at a dose of 10^6.5^ EID_50_. The mice lungs and nasal washes were collected on dpc 2 (*n* = 5) and 4 (*n* = 5). TCID_50_ assay was performed for four types of samples: (a) dpc2 lungs, (b) dpc4 lungs, (c) dpc2 nasal washes, and (d) dpc4 nasal washes. The results were expressed as mean ± SD. Statistical significance was calculated using a one-way analysis of variance (one-way ANOVA) ( ^*∗*^*P* ≤ 0.05,  ^*∗∗*^*P* ≤ 0.01, ^*∗∗∗*^*P* ≤ 0.001).

To examine the protective efficacy of CIV^SA1^ against CIV, all the vaccinated or PBS-inoculated mice (except mice in the N.C. group) were challenged with 10^6.5^EID_50_ dose of CIV at 2 weeks after 2nd vaccination. All experimental groups were euthanized to obtain lungs and nasal washes at dpc 2 and 4, and TCID_50_ assay was performed to determine residual virus titer. The CIV^SA1^ IN group was the only group among the challenged groups in which the virus was not detected in the lungs and nasal washes on dpc 2 (Figures 9(a) and 9(c)). It showed significantly lower lung titers compared to the Caniflu-max vaccine groups. Furthermore, the CIV^SA1^ IM group also exhibited significantly lower viral titer. As shown in Figures 9(b) and 9(d), we found that no virus was detected in any of the vaccine groups, despite there was no difference in lung viral titers of positive control between dpc 2 and dpc 4. These results showed that CIV^SA1^ LAIV candidates induce higher immunity and protective efficacy in mice against CIV compared to the commercial vaccine and, in addition, the results of the protection efficacy were correlated with that of immune response capabilities.

## 4. Discussion

The NS1 protein of influenza A virus plays a critical role in counteracting the host IFN system. IFNs are a group of signaling proteins that are produced by cells in response to viral infections, and they play a key role in activating the host immune system to fight off the virus [28]. Truncation of the effector domain of the NS gene can affect viral replication by altering the ability of the virus to evade the host immune response [15, 16, 29]. The effector domain of NS1 includes the PKR binding site, p85*β*-binding site, CPSF30-binding site, and PDZ domain, which act to evade the host antiviral immune response in a variety of ways. The inhibition of PKR, a crucial component of host antiviral response mediated by IFN and ISGs, occurred through direct binding between 123 and 127 amino acid residues of NS1 and PKR junction region [30, 31]. A host p85*β* protein, a regulatory subunit of phosphoinositide 3-kinase (PI3K) enzyme, has been shown to inhibit viral replication and regulate apoptosis [32, 33]. The 141–142aa and 164–167 aa of NS1, interacting with p85*β* protein, are essential for modulating the PI3K signal. The residues 144–186aa of NS1, binding motif to CPSF30 (cleavage and polyadenylation specificity factor), inhibit the cleavage and polyadenylation of mRNA and increase influenza A virus replication [34, 35].

The PDZ ligand motif in NS1, located at the last four C-terminal aa residues, plays an important role in signaling pathways: regulating the activities, trafficking membrane proteins, and maintaining cell polarity and morphology [36]. As shown in Figure 2, the NS gene of SA1 strain generates an early stop codon by frameshift and, consequently, lacks important binding sites in the effector domain, described above that help viral replication. The use of C-terminal truncation of NS1 as an LAIV has been demonstrated in pigs, chickens, and horses [29, 37, 38]. In those studies, the vaccinated animals showed protection and reduced clinical signs against challenges with wild-type viruses. Furthermore, influenza A virus with truncated NS1 was characterized by not only highly being attenuated but also showing higher humoral responses and protection efficacy compared to that with absent NS1 in vivo [39]. In the case of the SA1 strain, which has a naturally truncated NS gene, it was revealed that the virus showed significantly increased expression of antiviral and pro-inflammatory genes in vitro [16]. Furthermore, in a mouse model, the recombinant PR/8 virus containing the concerned truncated NS gene showed dramatically reduced virulence, and the level of IFN-g cytokines was statistically elevated [15].

We hypothesized that the naturally truncated NS gene might be a suitable target for the development of LAIV candidates. Thereby, we developed recombinant H_3_N_2_ CIV with the naturally truncated NS gene using reverse genetics, and the potential of this recombinant virus as a live attenuated H_3_N_2_ canine influenza vaccine was evaluated in vitro and in vivo. The recombinant H_3_N_2_ CIV was confirmed to be inserted with the naturally truncated NS gene by nucleotide sequence analysis. As shown in the growth kinetics data of Figure 3, there was no significant difference in viral replication in MDCK cells and SPF Eggs. This finding was in consistent with the previously reported studies, in vitro characteristics of rPR/8 × KYG^NS^ and influenza viruses with long deletions in the NS segment [15, 40]. Nevertheless, this finding is controversial to the previously reported studies of in vitro characteristics of the H_3_N_8_ A/Equine/Kyonggi/SA1/2011 (KG11), the original virus of the NS gene used in our study [16, 17]. Probably in vitro, the NS gene has other mechanisms besides evading host antiviral effects, and several factors may contribute to growth efficiency, similar to the previous result of recombinant PR/8 × KYG^NS^. Although the functional roles of viral factors contributing to growth efficiency in cells and eggs are needed to be further characterized, the result that the H_3_N_2_ canine influenza live attenuated vaccine candidate grows efficiently in MDCK cells and SPF eggs suggests that it will be helpful for vaccine production.

In contrast to the result of growth efficiency in vitro, the viral titer in both lungs and nasal wash samples from CIV^SA1^-infected mice were significantly lower than that from CIV-infected mice on dpi 4. To explain the possible association between viral replications in vivo and C-terminal truncated NS gene, mRNA level of IFN-associated signal, known to be antagonized by NS1, was measured. The levels of RIG-I mRNA in lungs from CIV^SA1^-infected mice were 27 times as high as those from CIV-infected mice on dpi 3. Similarly, the mRNA level of RIG-I downstream genes, IRF7, ISG15, IFN-beta, RANTES, IL-6, and IP-10 was also upregulated in lungs from CIV^SA1^-infected mice, compared to that from CIV-infected mice. In this study, we found that the C-terminal truncated NS1 protein was unable to efficiently suppress the transcription of the host RIG-I gene and downstream genes of the RIG-I pathway. The NS1 protein exists in a homodimer form with effector domain and RNA-binding domain, which contributes to multimerization. It is assumed that the impaired multimerization, which was affected by the truncated NS1 protein, was unable to suppress the transcription of RIG-I pathway signals [41]. Similarly, this result is in accordance with the previously reported study, the impairment of IFN-antagonizing activity by naturally truncated NS1 protein in vitro [16].

To investigate whether the significant decrease in lung viral titer and the properly functioning RIG-I pathway signal have an impact on the reduction of lung histological score, we conducted histopathological and immunohistopathological analysis in lungs of CIV or CIV^SA1^-infected mice at dpi 2 and 4, using H&E staining and NP-antibody staining. The histopathological analysis results showed a decrease not only in the histopathological sum-up score but also in the immunohistopathological score in the lungs of mice infected with CIV^SA1^, compared to mice infected with CIV. These histopathological findings showed that insertion of the truncated NS gene in the virus leads to reduced pathogenicity in mice. In this analysis, a similar result was shown in a previous report, showing mild histopathological signs in mice infected with recombinant PR/8 × KYG^NS^.

Based on the result that CIV^SA1^ caused attenuation when inoculated into mice, we investigated whether this recombinant virus could be a candidate for LAIV by conducting immunological assays in CIV^SA1^-inoculated mice and comparing its protection efficiency with commercial vaccine-inoculated mice. Through ELISPOT and FACS analysis, it was found that type 1 helper T cells, cytotoxic T cells, and regulatory T cells were increased in CIV^SA1^-inoculated mice compared with those infected with wild-type virus. These results suggested that the recombinant virus with truncated NS gene induces higher T-cell responses than the wild-type virus in mice.

In the mouse experiments evaluating the utility of SA1 as a live vaccine, it was observed that both IgG binding to CIV and IgG neutralizing to CIV were significantly higher in the live vaccine groups compared to the commercial vaccine groups (Figure 7). Additionally, when the live vaccine was administered intranasally, a higher production of IgG was induced compared to intramuscular administration. Mucosal IgA production was only observed in the intranasally administered live vaccine group. Similarly, the results of cellular immunity, as assessed by IFN-*γ* ELISPOT assay, showed that the intranasally administered live vaccine group induces the highest formation of effector T cells in the spleen and lung (Figure 8).

To evaluate the vaccine protective efficacy against wild-type virus, immunized mice groups were challenged with CIV on the 2 weeks after 2nd vaccination. According to the immunogenicity results, the intranasally administered live vaccine group exhibited the highest protective immunity on dpc2, with all mice in this group showing no detectable virus in both lungs and nasal washes. In contrast, the commercial vaccine groups did not protect against the virus in the early infection stage (dpc2), but in the later infection stage (dpc4), they showed no detectable virus in lungs compared to the mock vaccine group. According to these results, this live attenuated vaccine candidate showed higher immunogenicity and protection efficacy against wild-type virus compared to the commercial inactivated vaccine. Similar to this study, using a truncated NS gene to create an LAIV and evaluating its efficacy in mice, aligns with findings from other previous reports [39, 42]. In previous studies utilizing the truncated NS gene, it was exhibited that viral attenuation could be achieved, along with the induction of sufficient humoral immunity and protective efficacy in mice. In this current research, this LAIV induced significantly higher levels of both mucosal immunity and cellular immunity, in addition to humoral immunity, compared to the inactivated vaccine. Furthermore, when the live vaccine was administered intranasally, it was observed to generate higher immunogenicity and protective efficacy than when administered intramuscularly.

In comparison to the LAIV outstanding efficacy, the foremost concern regarding the utility of this vaccine pertains to the potential for pathogenic reacquisition. The currently approved LAIV is a reassortant strain, incorporating six vRNAs (PB2, PB1, PA, NP, M, and NS) from the cold-adapted temperature-sensitive master donor strain (ca A/AA/6/60) and two vRNAs (HA and NA) from predicted circulating flu strain. In the approved strain, various point mutations were introduced at multiple sites in vRNAs, serving to attenuate the virus and enhance safety by inhibiting the rapid generation of revertants and/or single-gene reassortant that has lost the temperature-sensitive characteristic. In this study, the truncated NS vRNA has a sufficiently large deletion, making the probability of pathogenic reacquisition through point mutations very low. However, NS vRNA single-gene reassortant has the potential to reacquire pathogenicity. This represents a significant concern of pathogenic canine influenza emergence. Therefore, we anticipate that enhancing the safety of future NS-based LAIV can be achieved by altering the cis-acting segment-specific packaging signals in the vRNAs, as revealed in a previous study. Furthermore, as the number of animal experiments and human trials related to NS-LAIV increases, we expect that NS-LAIV safety concerns will be alleviated.

This report is the first description of a LAIV for H_3_N_2_ CIV whose attenuation mechanism is based on the natural truncation NS1 protein. The recombinant virus showed attenuation while retaining immunogenicity and protective efficacy in mice, which can be a strong candidate of LAIV against H_3_N_2_ CIV. Based on this study, we are planning further research in dogs to investigate the immunogenicity and protection characterization of the LAIV candidate.

## Figures and Tables

**Table 1:** The primers used for the comparative experiment of mRNA expression levels between CIV^SA1^ and CIV in the RIG-I and RIG-I downstream genes.

Genes	Forward primer	Reverse primer
RIG-I	AAAGCCTTGGCATGTTACAC	GGCTTGGGATGTGGTCTACT
IFN-b	GTCAGAGTGGAAATCCTAAG	ACAGCATCTGCTTGGTTGAAG
ISG15	CGCAGATCACCCAGAAGATCG	TTCGTCGCATTTGTCCACCA
IRF7	CCCACGCTATACCATCTACCT	GATGTCGTCATAGAGGCTGTTG
RANTES	CCCCATATTCCTCGGACACC	CTTCTCTGGGTTGGCACACA
IL6	TACCACTTCACAAGTCGGAGGC	CTGCAAGTGCATCATCGTTGTTC
IP-10	GTGGCATTCAAGGAGTACCTC	TGATGGCCTTCGATTCTGGATT
GAPDH	ACTCCACTCACGGCAAATTC	TCTCCATGGTGGTGAAGACA

**Table 2:** Groups of mice experiment for efficacy evaluation.

Group	*n*	Vaccines (HAU, volume)	Route	Challenge (titer, volume)
Caniflu IM (2^8^)	28	CaniFlu-max (2^7^HAU, 50 *μ*l)	Intramuscular	CIV (10^6.5^ EID_50_/mouse, 30 *μ*l)
Caniflu IM (2^6^)	28	CaniFlu-max (2^6^HAU, 25 *μ*l)	Intramuscular	CIV (10^6.5^ EID_50_/mouse, 30 *μ*l)
CIV^SA1^ IN (2^6^)	28	CIV^SA1^ (2^6^HAU, 25 *μ*l)	Intranasal	CIV (10^6.5^ EID_50_/mouse, 30 *μ*l)
CIV^SA1^ IM (2^6^)	28	CIV^SA1^ (2^6^HAU, 25 *μ*l)	Intramuscular	CIV (10^6.5^ EID_50_/mouse, 30 *μ*l)
P.C.	23	PBS (25 *μ*l)	Intranasal	CIV (10^6.5^ EID_50_/mouse, 30 *μ*l)
N.C.	15	PBS (25 *μ*l)	Intranasal	PBS (30 *μ*l)

## Data Availability

The data used to support the findings of this study are included within the article and in the supplementary files.

## Supplementary Materials

Supplementary Materials Table S1: criteria for grading lung histopathological lesions and IHC. Table S2: lung histopathological and IHC scores of mice infected with CIV and CIV^SA1^. Figure S1: lung histopathological and IHC sum-scores of mice infected with CIV and CIV^SA1^. Figure S2: ELISA titer of serum IgG and BALF IgA in the vaccinated mice groups. Serum and BALF samples were collected from mice at 2 weeks after the 1st and 2nd vaccines. Vaccines and vaccine injection route (HI titer): Caniflu IM (2^8^), Caniflu IM (2^6^), CIV^SA1^ IN (2^6^), CIV^SA1^ IM (2^6^).
