# Corrigendum to “Development of Live Vaccine Candidates for Canine Influenza H3N2 Using Naturally Truncated NS1 Gene”

**DOI:** 10.1155/tbed/9796375

**Published:** 2025-09-02

J. Hwang, S-W. Yoon, E. Ga, et al., “Development of Live Vaccine Candidates for Canine Influenza H3N2 Using Naturally Truncated NS1 Gene,” *Transboundary and Emerging Diseases* 2024, no. 1 (2024): 1–16, https://doi.org/10.1155/2024/4335836.

In the article titled “Development of Live Vaccine Candidates for Canine Influenza H3N2 Using Naturally Truncated NS1 Gene,” some funding information was missed from the Acknowledgments section.

The corrected section appears below:

**Acknowledgments**

This study was financially supported by the Chonnam National University (Grant 2020-2073) and the National Research Foundation of Korea (NRF) grant funded by the Korea Government (MEST) (Grants NRF-2020R1C1C1008347 and NRF-2021M3E5E3083401).

We apologize for this error.

