# Supplementary material for: Development of Live Vaccine Candidates for Canine Influenza H3N2 Using Naturally Truncated NS1 Gene
Source: Transbound Emerg Dis. 2024 Mar 22;2024:4335836. doi: 10.1155/2024/4335836 (PMC12017229; doi:10.1155/2024/4335836)
Supplement: Supplementary Materials — Table S1: criteria for grading lung histopathological lesions and IHC. Table S2: lung histopathological and IHC scores of mice infected with CIV and CIVSA1. Figure S1: lung histopathological and IHC sum-scores of mice infected with CIV and CIVSA1. Figure S2: ELISA titer of serum IgG and BALF IgA in the vaccinated mice groups. Serum and BALF samples were collected from mice at 2 weeks after the 1st and 2nd vaccines. Vaccines and vaccine injection route (HI titer): Caniflu IM (28), Caniflu IM (26), CIVSA1 IN (26), CIVSA1 IM (26). [file 4335836.f1.doc]

| Score | Lung histopathology | IHC result (x100 field) |
| --- | --- | --- |
| 0 | No lesions | No positive cells |
| 1 | Minimal | 1-20 positive cells |
| 2 | Mild | 21-50 positive cells |
| 3 | Moderate | 51-100 positive cells |
| 4 | Severe | Over 100 positive cells |

**Supplemental Table 1.** Criteria for grading lung histopathological lesions and IHC

| Animal ID | Histopathological scores* | | | | | IHC scores* |
| --- | --- | --- | --- | --- | --- | --- |
|  | Necrosis of bronchial epithelial cells | Peribronchial and perivascular cuffing | Inflammatory cell infiltration in the alveoli | Alveolar hemorrhage | Atelectasis |  |
| dpi 2 pbs | 0 | 0 | 0 | 0 | 0 | 0 |
| dpi4 pbs | 0 | 0 | 0 | 0 | 0 | 0 |
| dpi2 CIV | 0 | 1 | 1 | 1 | 1 | 3 |
| dpi2 CIV^SA1^ | 0 | 1 | 0 | 1 | 0 | 2 |
| dpi4 CIV | 0 | 1 | 1 | 1 | 2 | 4 |
| dpi4 CIV | 0 | 1 | 2 | 1 | 2 | 4 |
| dpi4 CIV | 0 | 1 | 0 | 0 | 0 | 3 |
| dpi4 CIV^SA1^ | 0 | 1 | 0 | 0 | 0 | 2 |
| dpi4 CIV^SA1^ | 0 | 1 | 0 | 1 | 1 | 3 |
| dpi4 CIV^SA1^ | 0 | 1 | 0 | 0 | 0 | 3 |

**Supplemental Table 2.** Histopathological findings in the lungs. * 0 : no lesions or no positive cells; 1 : minimal or 1-20 postive cells; 2 : mild or 21-50 positive cells; 3 : moderate or 51-100 positive cells; 4 : severe or over 100 positive cells.


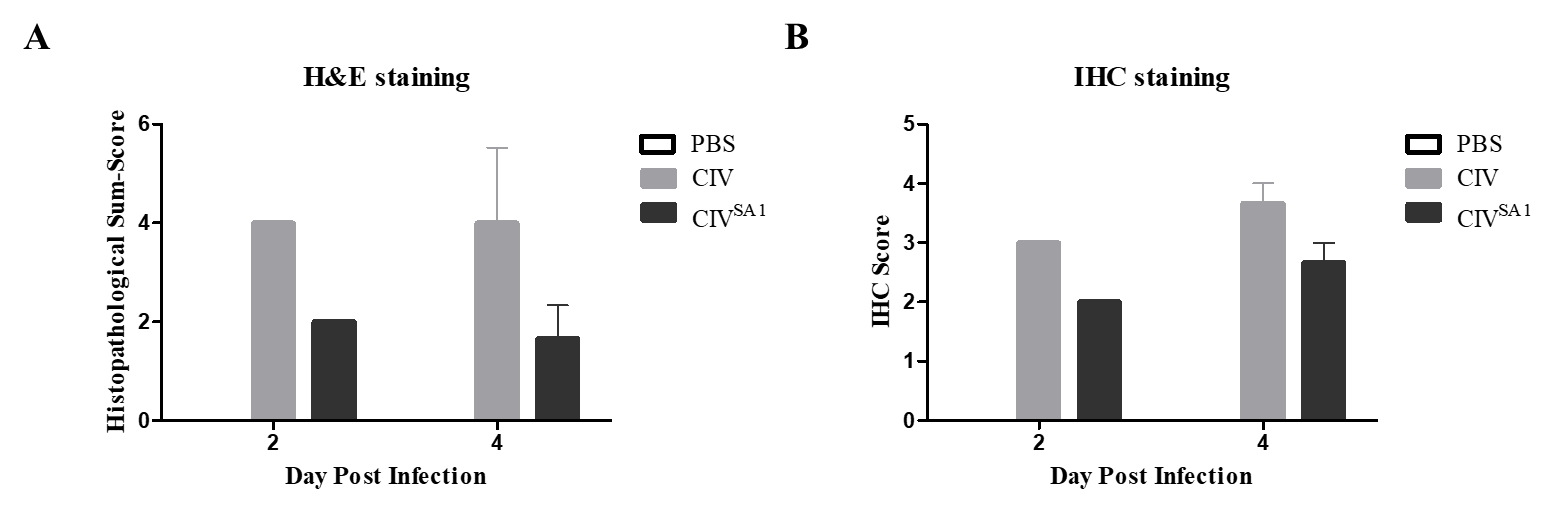


**Supplemental Figure 1.** Lung histopathological and IHC sum-scores of mice infected with CIV and CIV^SA1^. Mice were inoculated intranasally with CIV and CIV^SA1^ virus at a dose of 10^6.5^EID_50_ and each Lung tissues collected on dpi 2 (n=1) and 4 (n=3) were stained with H&E (A) and Influenza A NP antibody (B). The results were expressed as mean±SD.


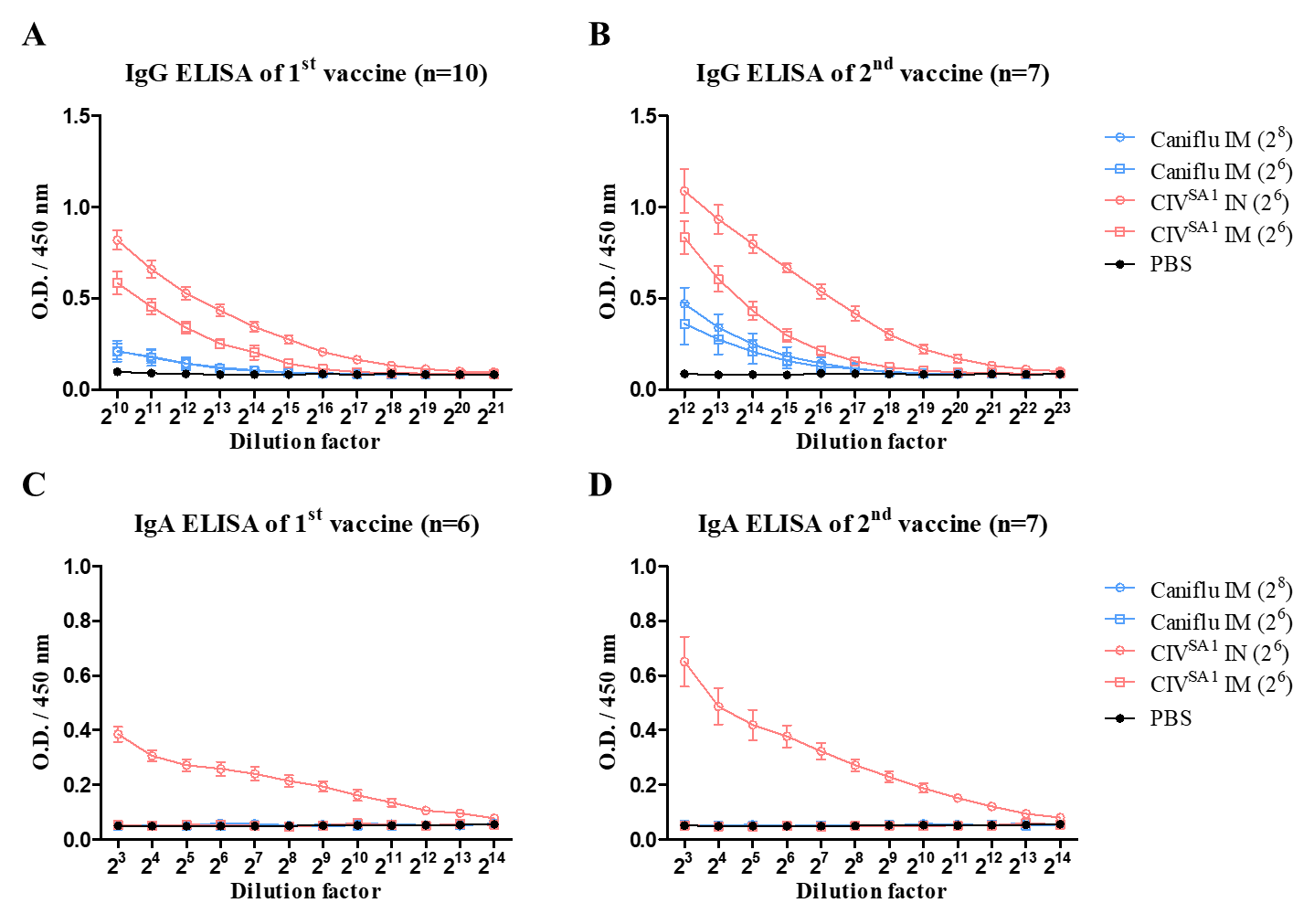


**Supplemental Figure 2.** Determining Serum IgG and BALF IgA antibody titer against CIV. The serum IgG and BALF IgA antibody titer was analyzed using ELISA coated with CIV whole viral particles. Serum samples were collected from mice at 2 weeks after the 1^st^ (A, C) and 2^nd^ vaccine (B, D). The samples were diluted as indicated in X axis, respectively. ELISA was performed by measuring the O.D. values at 450nm wavelength. The results were expressed as the mean±SD and the number of samples for each experiment was indicated in the respective figures.
